# Recent Progress in Hydrogel Synthesis and Biomedical Applications

**DOI:** 10.3390/gels11060456

**Published:** 2025-06-14

**Authors:** Luxing Wei, Jun Huang

**Affiliations:** Center for Advanced Jet Engineering Technologies (CaJET), Key Laboratory of High Efficiency and Clean Mechanical Manufacture of Ministry of Education, School of Mechanical Engineering, Shandong University, Jinan 250061, China

## 1. Introduction

**Figure 1:**
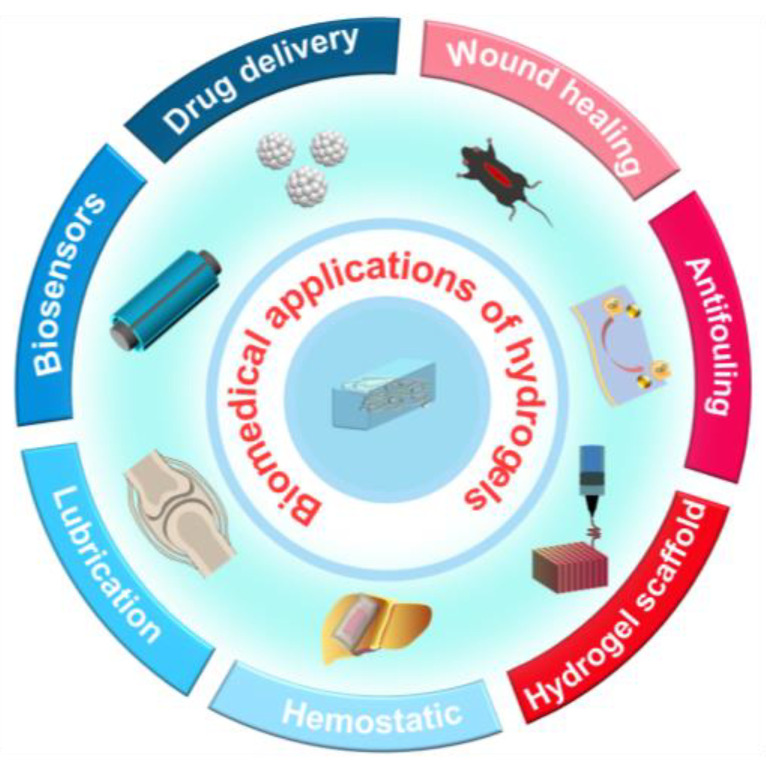
Conceptual diagram of hydrogel biomedical applications.

Hydrogels are three-dimensional network structures formed by hydrophilic polymer chains through chemical or physical cross-linking [1,2]. Their structure and properties are influenced by the composition of the polymers and the cross-linking methods between the molecular chains, resulting in a diverse range of characteristics. For instance, hydrogels exhibit tunable swelling/deswelling behavior, multifunctional stimuli-responsive behavior, and good mechanical performance, offering new research directions for the design and application of soft materials [3]. The high water content of hydrogels imparts both solid and fluid mechanical characteristics, with elastic moduli comparable to those of biological tissues. As shown in Figure 1, this unique combination enhances their biocompatibility and offers significant potential for biomedical applications, including drug delivery systems, tissue engineering scaffolds, and biological electrodes [4].

Based on their cross-linking mechanisms, hydrogels are classified into physically cross-linked, chemically cross-linked, and physically/chemically hybrid cross-linked types [5]. Chemical cross-linking primarily involves covalent bond formation between polymer chains, typically achieved via free radical polymerization. Although most chemical cross-linking is permanent, there are also reversible covalent cross-linking types, such as Schiff base bonds and boronate ester bonds [6]. Physical cross-linking relies on non-covalent interactions between molecules, including electrostatic interactions, hydrogen bonding, and hydrophobic interactions. The advantage of physical cross-linking lies in its reversibility. Following disruption to the hydrogel network, these non-covalent interactions can spontaneously reform under suitable conditions, endowing hydrogels with excellent self-healing capabilities and fatigue resistance. Hybrid physically/chemically cross-linked hydrogels integrate mechanisms to optimize both the mechanical and functional properties of the hydrogels through a synergistic effect [7]. Therefore, by designing novel monomers, developing controllable cross-linking methods, and constructing multifunctional composite networks, the physicochemical properties of hydrogels (such as degradation rate, mechanical strength, swelling behavior, and pore structure) can be precisely tuned to meet the technical requirements of complex biomedical applications.

**Figure 2:**
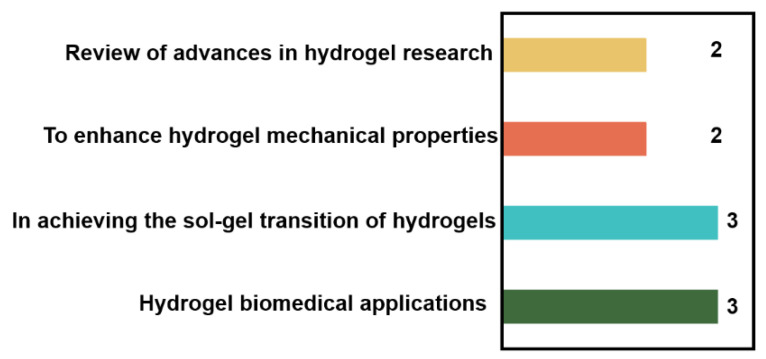
The themes and distribution of articles covered in this Special Issue.

In this context, the Special Issue, entitled “Synthesis and Applications of Hydrogels (2nd Edition)” in the journal *Gels* aims to gather contributions from leading scholars in the field. As shown in Figure 2, the Special Issue includes ten articles that explore the innovative applications and scientific developments of hydrogels in various domains. These studies cover a wide range of topics, including the design of novel cysteine–silver hydrogel composite systems, responsive anticancer hydrogels, hydrogel-based drug delivery systems, and advancements in antimicrobial hydrogel wound dressings and biomimetic materials, thereby encompassing multiple aspects of the fundamental research on and practical applications of hydrogels.

## 2. Contributions

**Figure 3:**
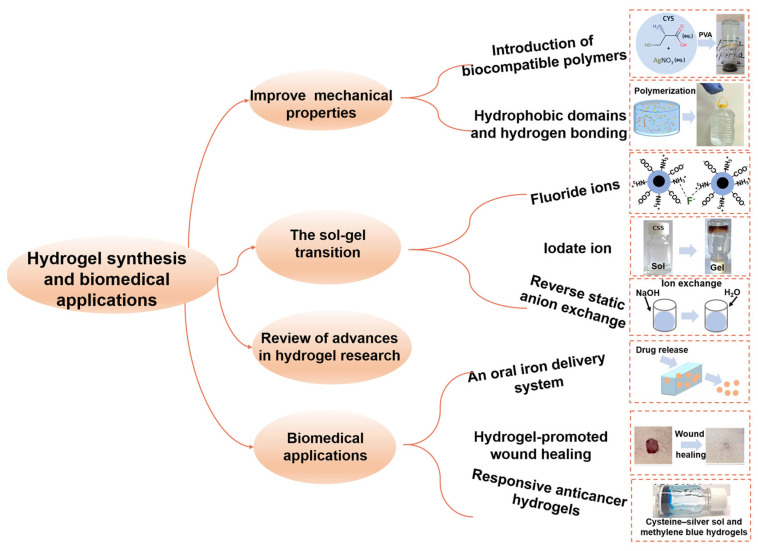
Summary of hydrogel synthesis and biomedical applications presented in this Special Issue of *Gels*.

Figure 3 provides a brief overview of the summaries related to hydrogel synthesis and their biomedical applications in this issue of *Gels*, presenting the core thematic framework of the Special Issue to the readers. The following sections will detail the distribution of the articles and introduce the research focuses and innovations of each paper, aiding readers in gaining a comprehensive understanding of the content of this Special Issue.

In their review of advances in hydrogel research, Jie et al. focus on the application of antimicrobial hydrogels in wound dressings, systematically detailing the research progress in inherent antimicrobial properties, the loading of functional agents, and novel therapeutic hydrogels [8]. The study demonstrates that innovative strategies such as pH-responsive designs, photothermal/photodynamic therapy, and metal–organic frameworks enhance the antimicrobial efficacy of hydrogels. Despite progress in the mechanical properties, antibacterial durability, and biocompatibility of existing hydrogel materials, challenges remain regarding large-scale production and effective synergy between multiple mechanisms. In addition, Maria et al. outline the cutting-edge developments in biomimetic materials, focusing on areas such as hydrogels, collagen-based composites, surface modification, and 3D bioprinting [9]. The article highlights that hydrogels offer unique advantages for tissue engineering due to their programmable stimulus responsiveness and extracellular matrix-like properties. The article also emphasizes that the clinical translation of biomimetic materials needs to overcome key bottlenecks, such as biosafety, while future applications will rely on the continuous advancement of interdisciplinary collaboration.

In their research paper, to improve the mechanical properties of hydrogels, Dmitry et al. introduce biocompatible polymers (e.g., polyvinyl alcohol (PVA)) to the L-cysteine–silver nitrate system, addressing the poor mechanical performance of low-molecular-weight gelling agents [10]. PVA interacts with the cysteine–silver sol via hydrogen bonding and hydrophobic interactions, endowing the hydrogel with excellent rheological properties and a porous structure (pore size of 2–10 μm). In vitro cytotoxicity tests indicate that the hydrogel is non-toxic to normal fibroblasts, making it suitable as a cell carrier or wound dressing in regenerative medicine. Additionally, Vukasin et al. prepare a poly(methacrylic acid) (PMA)/gelatin hydrogel, where the hydrophobic domains of gelatin and hydrogen bonding with PMA create permanent cross-linking points, resulting in outstanding mechanical properties (tensile strength of 1.44 MPa and compressive strength of 24.81 MPa) [11].

In achieving the sol–gel transition of hydrogels, Dmitry et al. demonstrate that fluoride ions (F^−^) can induce the sol–gel transition in the L-cysteine/silver nitrate system, while other halides (Cl^−^, Br^−^, and I^−^) lead to precipitation [12]. The hydrogel exhibits high toxicity towards human squamous carcinoma cells but shows lower toxicity to normal cells. The hydrogel’s anticancer activity provides a novel approach for the local treatment of malignant skin tumors. Additionally, Dmitry et al. reveal the mechanism of supramolecular gel formation in the cysteine–silver sol (CSS)–iodate ion (IO_3_^−^) system [13]. The study finds that IO_3_^−^ serves simultaneously as a gelling agent and a light-responsive agent. In dark conditions, IO_3_^−^ reacts with CSS through a redox reaction to generate silver iodide/silver oxide nanoparticles, forming a weak gel. Upon exposure to visible light, the gel network undergoes increased fiber formation, and the color changes from green-yellow to brown, accompanied by a significant increase in viscosity. Furthermore, Tim et al. propose a method for preparing highly basic diallyldimethylammonium hydroxide (DADMAOH) hydrogel particles through a reverse static anion exchange method [14]. The neutral diallyldimethylammonium chloride gel is mechanically fractured and suspended in a NaOH solution, where it undergoes reverse static anion exchange to generate DADMAOH particles with a residual halogen content of less than 0.3%.

In the design of functional hydrogel systems and their biomedical applications, Polina et al. develop an oral iron delivery system based on hydroxypropyl cyclodextrin (HPCD) and poly(methylsilsesquioxane) (PMSSO) hydrogels [15]. By forming a host–guest complex of ferrous gluconate with HPCD at a 1:1 molar ratio, and subsequently embedding it within a PMSSO hydrogel network, the sustained release of iron ions in the gastric environment is achieved. This system holds promise for improving adherence and efficacy in the treatment of anemia. Sarawut et al. embed cannabidiol-loaded lipid nanoparticles into a polyvinyl alcohol–sodium alginate composite hydrogel. In vitro experiments demonstrate that this hydrogel system promotes fibroblast migration (with a 48 h closure rate of 84.5%) and reduces intracellular reactive oxygen species levels [16]. In an in vivo rat model, this hydrogel significantly accelerates wound closure (with a healing rate of nearly 100% after 28 days). Histological analysis indicates that it improves healing quality by promoting epithelialization, collagen deposition, and angiogenesis. Furthermore, Dmitry et al. synthesize a composite photo-responsive hydrogel based on CSS and methylene blue (MB) for photodynamic therapy in cancer treatment [17]. The CSS and MB form a uniform network through non-covalent interactions, combining the anticancer activity of silver nanoparticles with the photodynamic effects of MB. In vitro experiments demonstrate that the hydrogel exhibits a synergistic cytotoxic effect against human squamous cell carcinoma, with activity enhanced by two to three times upon light exposure, while showing low toxicity to normal keratinocytes.

## 3. Conclusions

We anticipate that this Special Issue will provide readers with cutting-edge insights into the synthesis and application of hydrogels. This rapidly emerging field has attracted broad participation from multiple disciplines, including biology, chemistry, physics, and materials science. The ten articles included in this Special Issue showcase the diverse innovations in hydrogel research, covering topics from material design and response mechanisms to cross-disciplinary applications. Future research can focus on the integration of multiple responsive functions, the optimization of biosafety, and scalable production technologies to promote the translational application of hydrogels in precision medicine and smart devices.

Given the rapid development of this field, we recognize the limitations of covering all relevant topics within a single Special Issue. However, owing to their interdisciplinary nature and high quality, we believe that these articles provide a broad perspective on the latest advances in biomedical hydrogels.
